# Neural Network Direct Control with Online Learning for Shape Memory Alloy Manipulators

**DOI:** 10.3390/s19112576

**Published:** 2019-06-06

**Authors:** Alfonso Gómez-Espinosa, Roberto Castro Sundin, Ion Loidi Eguren, Enrique Cuan-Urquizo, Cecilia D. Treviño-Quintanilla

**Affiliations:** 1 Tecnologico de Monterrey, Escuela de Ingeniería y Ciencias, Ave. Epigmenio González 500, Fracc. San Pablo, Querétaro 76130, Mexico; cdtrevino@tec.mx; 2 KTH Royal Insitute of Technology, 114 28 Stockholm, Sweden; rosun@kth.se; 3 Escuela Politécnica Superior, Universidad Mondragón, 20500 País Vasco, Spain; ion.loidi@alumni.mondragon.edu

**Keywords:** shape memory alloys, artificial neural networks, control, manipulators

## Abstract

New actuators and materials are constantly incorporated into industrial processes, and additional challenges are posed by their complex behavior. Nonlinear hysteresis is commonly found in shape memory alloys, and the inclusion of a suitable hysteresis model in the control system allows the controller to achieve a better performance, although a major drawback is that each system responds in a unique way. In this work, a neural network direct control, with online learning, is developed for position control of shape memory alloy manipulators. Neural network weight coefficients are updated online by using the actuator position data while the controller is applied to the system, without previous training of the neural network weights, nor the inclusion of a hysteresis model. A real-time, low computational cost control system was implemented; experimental evaluation was performed on a 1-DOF manipulator system actuated by a shape memory alloy wire. Test results verified the effectiveness of the proposed control scheme to control the system angular position, compensating for the hysteretic behavior of the shape memory alloy actuator. Using a learning algorithm with a sine wave as reference signal, a maximum static error of 0.83° was achieved when validated against several set-points within the possible range.

## 1. Introduction

Nonlinear systems have been an active research area over the past few decades, partly fueled by the needs of modern industry. As new actuators and materials are incorporated into industrial processes, additional challenges can be posed by their sometimes complex behavior. An example of this is the nonlinear hysteresis commonly found in shape memory alloys (SMAs) [1], certain nanocomposite materials [2], and micro-electromechanichal systems (MEMS) [3].

In applications where hysteresis behavior is present, the inclusion of a suitable hysteresis model in the control system design allows the controller to have better tracking of the system and may result in a reduced error response of the controlled variable [4,5,6]. The methods that have been used to model these behaviors are many, and include artificial neural networks (ANNs) [4], elliptical approximations of hysteresis loops [5], the Preisach model [6], ideal order hexagonal arrays adjusted using the Monte Carlo method [7], a modified Prandtl–Ishlinskii Model [8], and the Semilinear Duhem Model [9].

Although the use of a suitable model allows a better control of a hysteretic system, a major drawback is that each system responds in a unique way, meaning that the controllers characteristics, such as effectiveness, speed, number of cycles, etc., may vary from one system to another, even if the control techniques are the same [10,11]. As an example, second-order models have been used to analyse and model output signal sensors. However, this process is time-consuming, and when completed, the resulting algorithm can only be applied successfully to the system it was specifically designed for [10]. Another previously used method for approximating and tracking the performance of a system, is to make use of ANNs. This has been done, for instance, for an inverted pendulum with an actuator displaying hysteretic behavior [11], and for an active magnetic bearing system [12]. Although ANNs are still under development in order to increase their accuracy in control system applications, they are frequently implemented due to their low computational cost [13].

Neural networks (NNs) have demonstrated their ability to identify, and compensate for, hysteresis in systems in which high precision is mandatory [14,15,16,17]; an adaptive wavelet NN controller with estimation of the friction force hysteresis was proposed for a piezo positioning mechanism [14], an ANN to identify, and compensate for, gear backlash hysteresis was tested for a precision position mechanism [15], a radial basis function neural network and a sliding control scheme was proposed for motion tracking control of piezoelectric actuators [16], and a dynamic recurrent NN with a proportional-derivative (PD) controller was implemented to identify and control a magneto restrictive actuator [17].

SMAs are recognized as promising smart materials for creating compact, high power-to-weight ratio actuators. However, position control of SMA actuators represents a major challenge for practical applications due to their nonlinear hysteretic behavior, producing steady-state errors when conventional controllers are used [18]. To overcome this problem, proportional-integral-derivate (PID) controllers with hysteresis models have been implemented [18,19,20]; for position control of SMA actuators using the generalized Prandtl–Ishlinskii inverse model [18], for micro-positioning control of SMA actuators by modeling the hysteresis using NNs [19], and for magnetic SMA actuators by using a radial basis function NN to obtain the Jacobian information of the system in order to adjust the controller parameters [20]. In addition, neural network controllers, previously trained to identify the system were proposed to control SMA actuators [21,22,23,24]; using the inverse of the ANN that replicate the dynamics of the SMA force actuator to implement the controller [21], implementing a model predictive controller based on a functional link ANN to control the linear memory metal actuator displacement [22], and realizing a recurrent neural model predictive, variable structure, controller designed to control a one degree of freedom (1-DOF) rotary manipulator actuated by an SMA wire [23,24]. The main disadvantages of these recurrent neural network controllers are the complexity of the control system, and the requirement of training the neural network, to identify the system, prior to the implementation of the control. During the identification of the system, a neural network captures the model of the plant. Later this plant model is utilized for the design of the controller. The control signal is only delivered to the plant, after these two stages are completed. Previous inputs/outputs data are gathered from the operation of the plant, and are utilized to train the neural network model, offline in batch mode [23]. Previous information from the controller outputs and from the plant outputs are the inputs utilized for the neural plant model, implementing a recurrent neural network structure [24].

In this work, a neural network direct control, with online learning, is developed for position control of shape memory alloy manipulators. The developed network consists of three inputs, four hidden layer perceptrons and one output, all using a sigmoidal activation function. In contrast to previous works, weight coefficients are updated online by using the actuator position data while the controller is applied to the system, without previous training of the neural network weights nor the inclusion of a hysteresis model. A real-time, low computational cost control system was implemented. Experimental evaluation was performed on a 1-DOF manipulator system actuated by a shape memory alloy wire and test results verify the effectiveness of the proposed control scheme to control the system angular position, compensating for the hysteretic behavior of the shape memory alloy actuator.

The remainder of the paper is organized as follows: Hysteresis nonlinearity is described in Section 2.1. Shape memory alloy nonlinear behavior is introduced in Section 2.2. Section 2.3 presents the NN direct controller with online learning using back-propagation. Section 3 describes the experimental setup and the results are presented in Section 4. Finally, in Section 5, concluding remarks are provided.

## 2. Materials and Methods

### 2.1. Hysteresis

**Figure 1:**
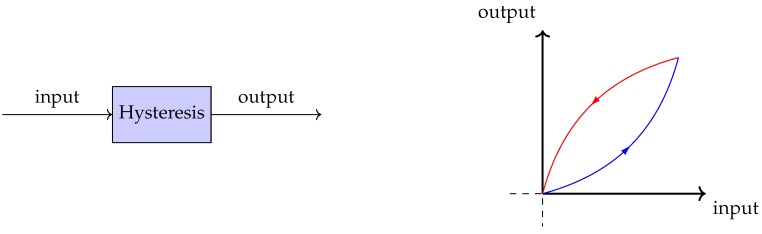
Typical input-output relation for a system with hysteresis.

Hysteresis is a strongly nonlinear phenomena, where *strongly* indicates that it cannot be linearized. The non-ability of being linearized is a consequence of the *memory effect* in hysteretic systems, which means that the output of the system is dependent not only on the current input, but also on the previous state of the system [6,9]. Hysteresis can be found in many different areas, including structural mechanics, aerodynamics, and electromagnetics [9]. Because of the memory effect, an input–output mapping for a system with hysteresis seldom is *injective*, since one input often may result in two distinct outputs depending on the system history (illustrated in Figure 1). This of course implicates that hysteresis cannot be modeled in the sense of an ordinary mathematical function, but requires a more sophisticated framework. One of these frameworks was proposed by Preisach as a means to model the hysteresis found in magnets, and was further improved upon by mathematician Krasnoselskii to become what is now referred to as the Preisach model [25].

#### The Classic Preisach Model

**Figure 2:**
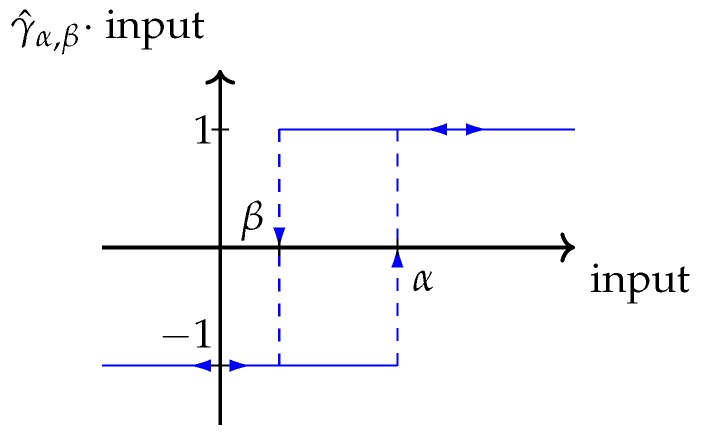
Definition of the fundamental hysteresis operator ${\hat{\gamma }}_{\alpha \beta }$. When the input is in the range (α,β), the output is positive if the input reached this range from above and negative if it was reached from below.

The model is most often represented by the *Preisach operator* $\hat{\Gamma }$, which can be expressed as a double integral of variables α,β

(1) $$\hat{\Gamma }=\underset{\alpha \geq \beta }{\int \int }\mu \left(\alpha ,\beta \right){\hat{\gamma }}_{\alpha \beta }d\alpha \;d\beta ,$$

where μ(α,β) is a weight function called the *Preisach function*, and ${\hat{\gamma }}_{\alpha \beta }$ is a fundamental hysteresis operator, often called *hysteron*. The hysteron is a mapping onto {−1,1} with memory properties as seen in Figure 2 [26]. Using the Preisach operator, systems with hysteresis, like the one in Figure 1, can now be modeled by finding the correct weight function μ(α,β).

### 2.2. Shape Memory Alloys

**Figure 3:**
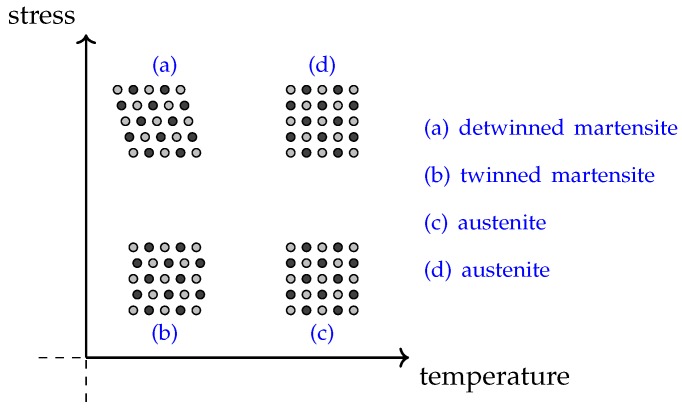
Stress–temperature plane showing schematic crystal structures of the different phases of a typical SMA.

Shape memory alloys, or SMAs, receive their name from their property of exerting a force to return to a certain *memorized* shape when heated, giving them the ability to convert heat into mechanical energy [23]. This effect is due to SMAs having two solid phases; a high temperature phase called *austenite* and a lower temperature phase called *martensite*, differentating themselves by having different crystal structures [24]. The austenite form is typically cubic and rigid, while the martensite usually is tetragonal, orthorhombic or monoclinic, giving it the possibility to change shape (e.g., stretch) upon being subjected to a force. Because of this, it is common to distinguish between the unstretched *twinned martensite* form and the stretched *untwinned* one. The relationship between the different phases can be seen in the stress–temperature-plane in Figure 3, for which it is also important to mention that the stress is sufficiently small to allow austenite form even under stress conditions when temperature is increased (seen in case (d)). For higher stress levels the austenite form could not be achieved even when increasing the temperature, but would rather stay in its detwinned martensite form (not shown in this figure) [27].

**Figure 4:**
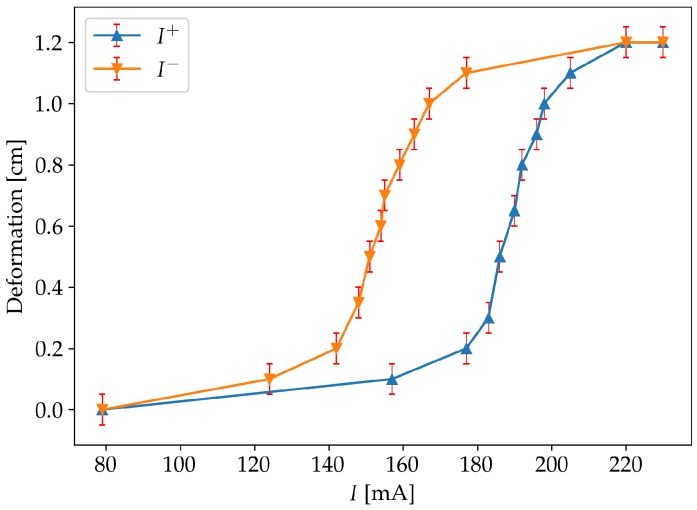
Plot showing deformation versus current for a Flexinol wire. $I^{+}$ denotes that the current is monotonically increasing, while $I^{-}$ denotes a monotonical decrease.

The transition between the untwinned martensite form and the austenite is highly hysteretic, as shown by the experimental results for a Flexinol SMA wire in Figure 4. The wire was 310 mm long, Ø 0.13 mm and had a 50 g weight suspended to it.

### 2.3. Neural Networks

#### 2.3.1. Feedforward Neural Networks with Sliding Window

In accordance with [28], a multilayer recurrent neural network is an extension of the classic perceptron that allows for more possibilities when it comes to the desired behavior, being able to approximate dynamic systems. The increased functionality comes from incorporating a number of so-called *hidden layers* in between the input and output layers, from a learning process called *back-propagation* and from making use of previous input/output values (i.e., recurrent neural network). Recurrent neural networks can approximate dynamic systems, represented by differential equations, however, feed forward neural networks can only approximate algebraic functions. Conventional recurrent neural networks incorporate temporal information in the hidden layer, by using previous output values as inputs for this same layer. On the other hand, feedforward neural networks with sliding windows can be implemented using previous input/output values as inputs for the input layer.

**Figure 5:**
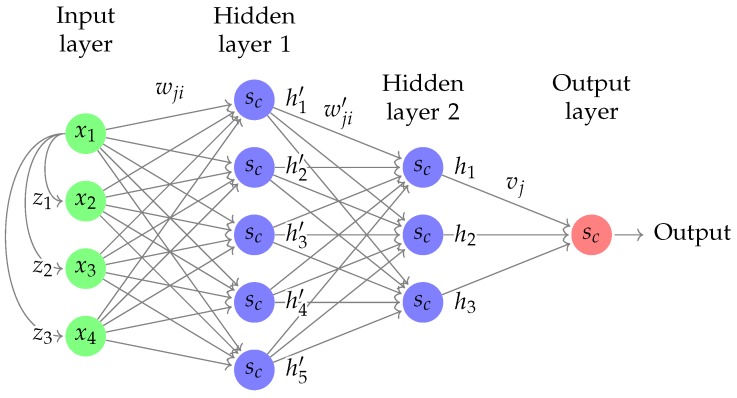
Schematic of a neural network with two hidden layers and a four element sliding window, where $s_{c}$ denotes the sigmoid activation function and $w_{ji}$, $w_{ji}^{\prime }$, $v_{j}$ are weight coefficients.

A hidden layer consists of a certain amount of perceptrons $h_{1},\ldots ,h_{m}$, each one connected to all of the perceptrons (or inputs) $h_{1}^{\prime },\ldots ,h_{n}^{\prime }$ in the preceding layer. Each of these connections are weighted by the coefficients $w_{ji}^{\prime }$, where subscripts j,i indicate the connection between perceptrons/inputs $h_{j}$ and $h_{i}^{\prime }$. A general illustration is shown in Figure 5, where the input layer uses present values as well as previous values $z_{1}$, $z_{2}$, $z_{3}$ for a feedforward neural network structure with a sliding window.

For a neural network with one hidden layer and a single output, the inputs can be defined as $x_{i}$, the hidden layer perceptrons as $h_{j}$ and the weights as follows

$w_{ji}$    the weight of the connection between input $x_{i}$ and hidden layer perceptron $h_{j}$
$v_{j}$    the weight of the connection between hidden layer perceptron $h_{j}$ and output perceptron.
  it has been regarded as items, please confirm

#### 2.3.2. Back-Propagation

Following the methodology of [28,29], the back-propagation algorithm can be used to train a multilayer neural network into reaching a desired behavior. This is done by iteratively changing the weights until the error is reduced to an acceptable level. A solution is thus any collection of weights $w_{ji},v_{j}$ that are able to achieve this. The back-propagation algorithm finds these weights by making use of the method of *gradient descent*. Using gradient descent the aim is to minimize the error function

(2) $$E=\frac{1}{2}\sum_{i}{\left|e_{i}\right|}^{2}=\frac{1}{2}\sum_{i}{\left|y_{ri}-y_{i}\right|}^{2}.$$

The gradient ∇E is only defined if *E* is continuous and differentiable, and thereby, the sigmoid $s_{c}:R\to \left(0,1\right)$ given by

(3) $$s_{c}\left(x\right)=\frac{1}{1+e^{-cx}},$$

will be used as activation function, where *c* can be chosen arbitrarily and decides the steepness of the curve.

As a means of increasing the flexibility of the activation function, a so-called *bias*, θ, can be used. The role of the bias is to shift the activation function $s_{c}\left(x\right)$ in the positive or negative direction of the *x*-axis. A simple way of adding the bias is to expand the input vector $\left(x_{1},\ldots ,x_{n}\right)$ with 1, to get $\left(x_{1},\ldots ,x_{n},1\right)$ and the weight vector $w=(w_{1},\ldots ,w_{n})$ with −θ, to get $w=(w_{1},\ldots ,w_{n},-\theta )$. This permits the comfortable notation of a bias-shifted perceptron as

(4) $$s_{c}\left(\sum_{i}w_{i}\;\cdot \;x_{i}-\theta \right)=\frac{1}{1+\exp[-c\left(\sum_{i}w_{i}\;\cdot \;x_{i}-\theta \right)]}=s_{c}\left(w\cdot x\right).$$

#### 2.3.3. Calculation of the Gradient

**Figure 6:**
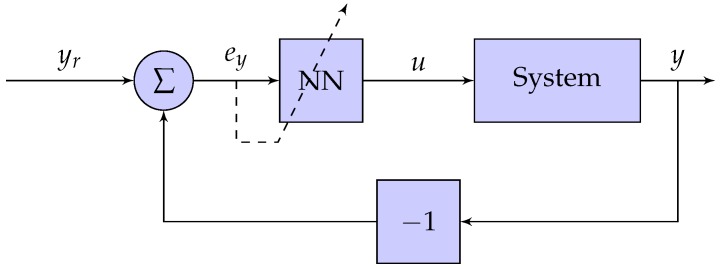
A block scheme of the control system.

For a neural network with one hidden layer and a single output, the gradient can be calculated in a straightforward manner using chain rule and the configuration of the control system, as seen in Figure 6 [28].

Since the weights $w_{ji},v_{j}$ are the ones to be adjusted, the gradient is calculated with respect to these. Therefore, the gradient is defined

(5) $$\nabla E=\left[\begin{array}{c} \frac{\partial E}{\partial v_{j}}\\ \frac{\partial E}{\partial w_{ji}} \end{array}\right],$$

with its partial derivatives

(6) $$\begin{array}{c} \frac{\partial E}{\partial v_{j}}=\frac{\partial E}{\partial e_{y}}\frac{\partial e_{y}}{\partial e_{u}}\frac{\partial e_{u}}{\partial u}\frac{\partial u}{\partial r}\frac{\partial r}{\partial v_{j}}=-e_{y}u\left(1-u\right)\frac{\partial e_{y}}{\partial e_{u}}h_{j} \end{array}$$

(7) $$\begin{array}{c} \frac{\partial E}{\partial w_{ji}}=\frac{\partial E}{\partial e_{y}}\frac{\partial e_{y}}{\partial e_{u}}\frac{\partial e_{u}}{\partial u}\frac{\partial u}{\partial r}\frac{\partial r}{\partial h_{j}}\frac{\partial h_{j}}{\partial S_{j}}\frac{\partial S_{j}}{\partial w_{ji}}=-e_{y}u\left(1-u\right)v_{j}h_{j}\left(1-h_{j}\right)\frac{\partial e_{y}}{\partial e_{u}}x_{i}, \end{array}$$

where the relations

(8) $$\begin{array}{c} r=\sum_{j}v_{j}h_{j} \end{array}$$

(9) $$\begin{array}{c} S_{j}=\sum_{i}w_{ji}x_{i}, \end{array}$$

along with the definitions seen in Figure 5 and Figure 6 have been used, and where $e_{u}$ denotes the error between the current control signal and the control signal required to control the system. Note that, with the lack of a mathematical model of the system, the error $e_{y}$ cannot be expressed in analytical terms. Consequently, its partial derivative ∂*e_y_*/∂*e_u_* found in Equations (6) and (7), is unknown.

With the gradient explicitly calculated, the weights can now be adjusted iteratively as follows

(10) $$\begin{array}{c} v_{j}^{\left(t+1\right)}=v_{j}^{\left(t\right)}-\eta \frac{\partial E}{\partial v_{j}}=v_{j}^{\left(t\right)}+\eta h_{j}\delta^{1}\frac{\partial e_{y}}{\partial e_{u}} \end{array}$$

(11) $$\begin{array}{c} w_{ji}^{\left(t+1\right)}=w_{ji}^{\left(t\right)}-\eta \frac{\partial E}{\partial w_{ji}}=w_{ji}^{\left(t\right)}+\eta x_{i}\delta_{j}^{2}\frac{\partial e_{y}}{\partial e_{u}} \end{array}$$

where η represents the learning rate and $\delta^{1}=e_{y}u\left(1-u\right),\;\delta_{j}^{2}=\delta^{1}v_{j}h_{j}\left(1-h_{j}\right)$.

Considering that

(12) $$\frac{\partial e_{y}}{\partial e_{u}}=\mathrm{sgn}\left(\frac{\partial e_{y}}{\partial e_{u}}\right)\cdot \left|\frac{\partial e_{y}}{\partial e_{u}}\right|$$

and letting *η*·|∂*e_y_*/∂*e_u_*|→*η*, Equations (10) and (11) simplify to

(13) $$\begin{array}{c} v_{j}^{\left(t+1\right)}=v_{j}^{\left(t\right)}+\mathrm{sgn}\left(\frac{\partial e_{y}}{\partial e_{u}}\right)\eta h_{j}\delta^{1} \end{array}$$

(14) $$\begin{array}{c} w_{ji}^{\left(t+1\right)}=w_{ji}^{\left(t\right)}+\mathrm{sgn}\left(\frac{\partial e_{y}}{\partial e_{u}}\right)\eta x_{i}\delta_{j}^{2}, \end{array}$$

where sgn(∂*e_y_*/∂*e_u_*), in accordance with [29], can be found experimentally for the system.

The previously described neural networks suffice when we seek to approximate static functions. However, when the aim is to approximate a dynamic function, it is necessary to include the concept of time. This can be done by incorporating previous input/output values. For example, if u(t) is the output at a time *t*, then an arbitrarily large collection of previous output values u(t−T),u(t−2T),…,u(t−k·T), where *T* is the discretization time step and k∈N, can be tracked. Using these previous output/input values as inputs, the neural network can obtain further understanding of the system dynamics and, for instance, know whether the output is increasing or decreasing, if the time derivate of the output is increasing or decreasing, etc.

## 3. Experimental Setup

A 1-DOF manipulator actuated by a Ø 0.13 mm Flexinol wire (DYNALLOY, Inc., 1562 Reynolds Ave., Irvine, CA, USA) was controlled using a pulse-width modulated current with a maximum of 230 mA that was fed by an Agilent e3631A power supply (5301 Stevens Creek Blvd., Santa Clara, CA, USA) set to an 8 V limit. The system controller consisted of a National Instruments myRIO-1900 (National Instruments Corporation, 11500 North Mopac Expy, Austin, TX, USA), whose 12-bit PWM output was connected to all eight amplifiers of a ULN2803 Darlington transistor array (Toshiba, 1-1, Shibaura 1-chome, Minato-ku, Tokyo, Japan) and a PDB181-K420K-103B potentiometer (Bourns, Inc., 1200 Columbia Ave., Riverside, CA, USA). Several controlled parameters of the setup, including the weight of the manipulator arm and the maximum current allowed, were set so as to allow for repeatability, ensuring that the Flexinol wire was deformed reversibly [27]. With the current setup it was verified that this meant keeping the maximum strain below 4%. In our case, no two-way shape memory effect (SME) was observed and it was thus necessary to apply stress to the Flexinol wire in order to achieve any deformation.

**Figure 7:**
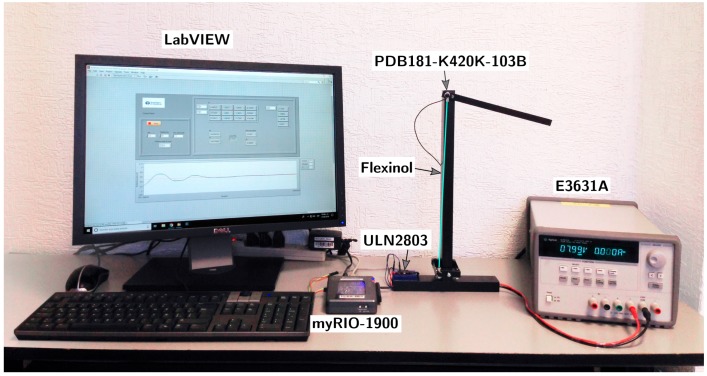
Photograph showing the experimental setup. The Flexinol wire has been marked in cyan.

To ensure the correctness of the angular position of the 1-DOF manipulator, the system was calibrated using an OptiTrack–Motive (NaturalPoint, Inc., 3658 SW Deschutes Street, Corvallis, OR, USA) motion capture system in a 16-cameras setup at a 240 Hz sampling rate (Appendix A). Figure 7 shows a photograph of the experimental setup. The dimensions of the manipulator are included in Appendix B.

**Figure 8:**
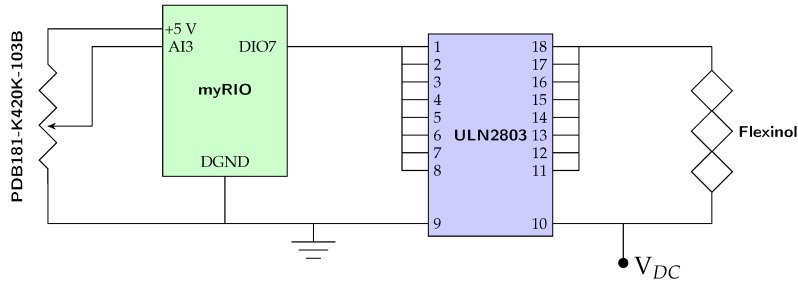
The circuit layout.

The output signal from the potentiometer was fed into a feedback loop through the myRIO-1900 analog input with 12 bits of resolution, as seen in Figure 6. The complete experimental setup circuit is illustrated in Figure 8.

The wire used as an actuator was made of Flexinol—a nickel-titanium alloy that exhibits SMA properties. Information from the manufacturer is seen in Table 1.

**Figure 9:**
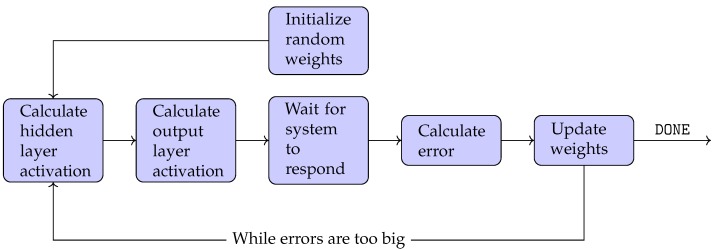
Flowchart illustrating the back-propagation algorithm.

The software used to program the myRIO-1900 was NI LabVIEW 2017 (National Instruments Corporation, 11500 North Mopac Expy, Austin, TX, USA). A feedforward neural network with a three element sliding window (initialized to zero), consisting of 3 inputs, 4 hidden layer perceptrons and one output perceptron, was implemented and trained online using the back-propagation algorithm, without any prior training or knowledge of the system. The three utilized inputs, which were the present and two previous values of the output system error, were computed as the difference between the reference output value $y_{r}$ to the measured output value *y*. Weights were adjusted for each iteration, until an acceptable error was reached. When no further adjustment was required, the learning procedure could be stopped. Optimization of the neural network architecture was made by performing experiments starting with a low number of hidden layer perceptrons and then increasing them one by one until no improvement of performance could be found in comparison to earlier experiments. The learning algorithm can be found in its entirety in Appendix C and is illustrated in the flowchart in Figure 9. The sample time of the controller was set to 20 ms and the PWM frequency to 60 Hz.

## 4. Results

**Figure 10:**
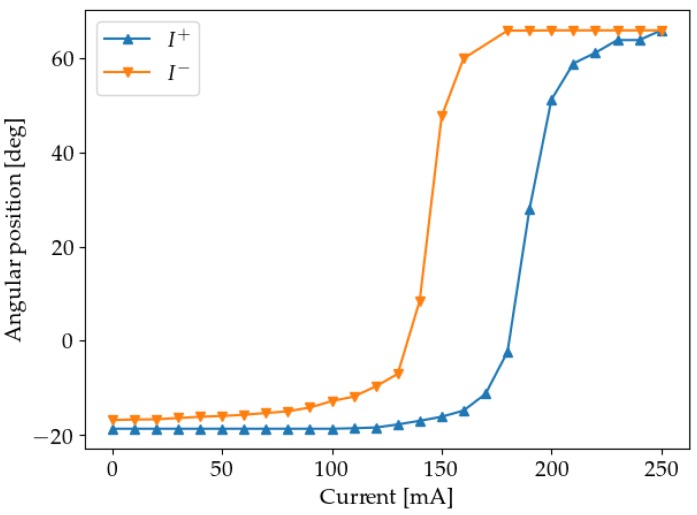
Characterization of the system. $I^{+}$ denotes that the current is monotonically increasing, while $I^{-}$ denotes a monotonical decrease

Prior to the experiments, a qualitative characterization of the system was performed. The results, confirming its hysteretic behaviour, can be seen in Figure 10.

Throughout every performed experiment, the Flexinol wire diamater as well as its original length and the ambient temperature remained constant. This is important since any change could affect the system behaviour [30].

**Figure 11:**
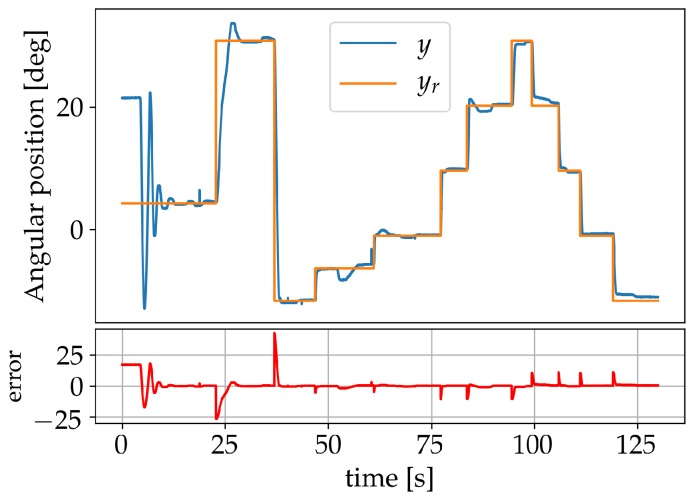
Learning session using set-points.

The neural network coefficients were adjusted online by sending the manipulator to different set-points within the possible range, starting with the learning coefficient η=2, a value deemed appropriate by trial and error. The set-point was changed as soon as a low-error value was achieved and η reduced by a factor of 2 each time. The reduction factor 2 was also found by trial and error and corresponded to a value for which the weights were updated just enough to learn the new set-point without affecting the previously learnt ones. Finally, η was set to zero, thereby terminating the learning process. Online learning allows the controller to continue improving its performance for additional set-points and adapting to changes produced by system degradation and external disturbances. The learning process was terminated to evaluate the capability of the controller, limited to the knowledge acquired during the online learning, when being tested for set-points changes and load disturbances. It should, however, also be pointed out that the controller performed just as well—or even better—when η was kept bigger than zero, thus never terminating the learning. Results during a learning session, for the reference angular position $y_{r}$, the measured angular position *y* and the output system error (marked beneath in red), are presented in Figure 11.

**Figure 12:**
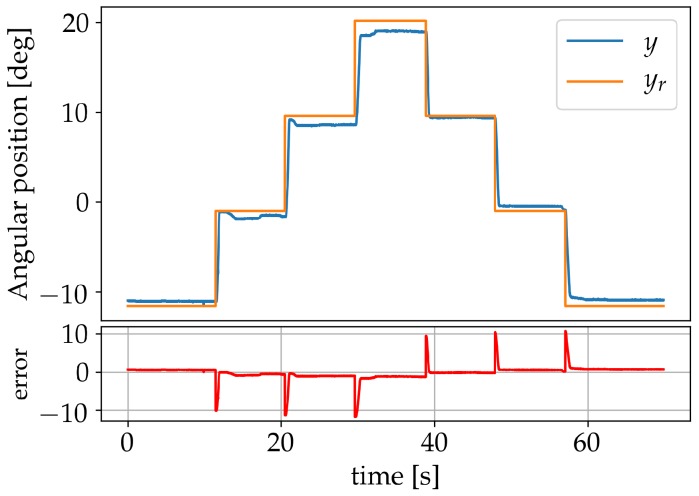
Results after learning using set-points.

With η set to zero, the response of the system was verified for set-points −12°, −1°, 10°, and 20°, as seen in Figure 12. The maximum static error achieved was 1.28°.

**Figure 13:**
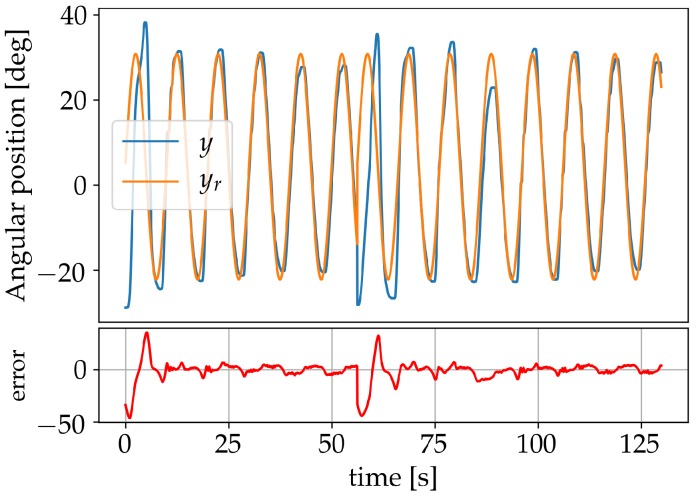
Learning session using a sine wave as reference signal.

**Figure 14:**
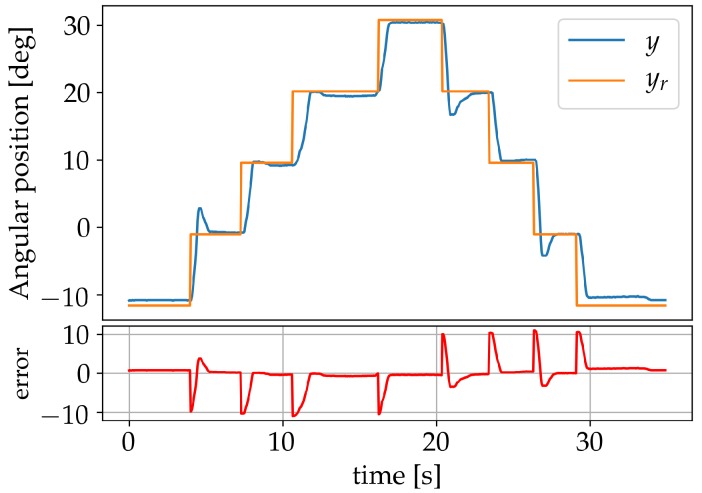
Results after learning.

A second learning procedure was implemented using a 0.01 Hz sine wave—ranging from −20° to 31°—as reference signal during learning. The learning coefficient η was primarily set to a value of 4 and gradually lowered to zero as the system started responding better to the reference signal. The learning process is illustrated in Figure 13. A validation done in the previous manner for set-points −12°, −1°, 10°, 20°, 31° can be seen in Figure 14 and resulted in a maximum static error of 0.83° for the set-point −1°. This error is reduced to 0.49° if we exclude the aforementioned set-point.

**Figure 15:**
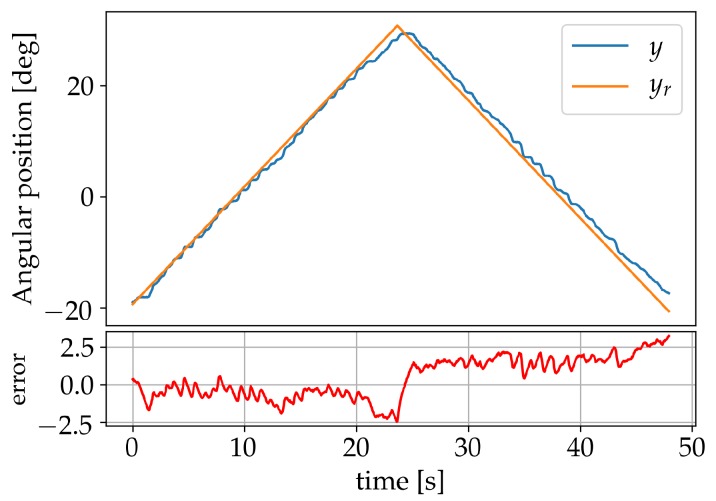
Results for a triangle wave form.

**Figure 16:**
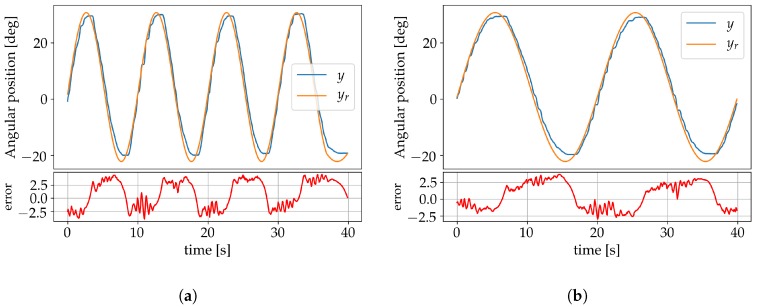
(**a**) Results for a 0.01 Hz sine wave; (**b**) Results for a 0.005 Hz sine wave.

**Figure 17:**
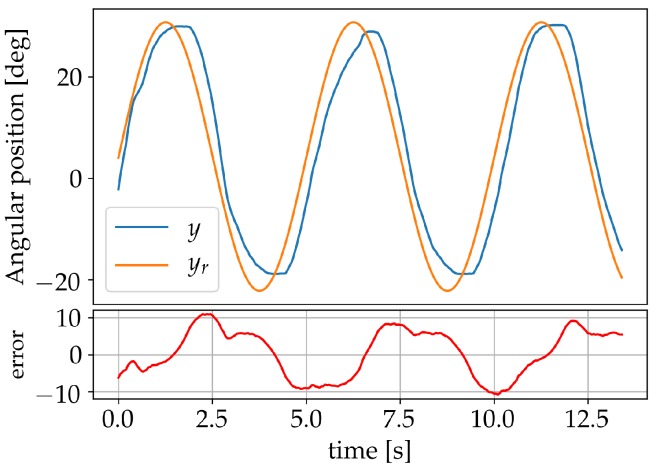
Result for a 0.05 Hz sine wave.

Further validation of the system performance after learning was made using triangular and sine wave forms as reference signals. The triangle wave form was set to a frequency of 0.04 Hz with an amplitude of 53° and the results are shown in Figure 15. The sinusoidals were set to an amplitude of 42° and to frequencies 0.005 Hz, 0.01 Hz and 0.02 Hz. Figure 16a shows the result for the learning frequency of 0.01 Hz and displays no significant phase lag. In Figure 16b the result for the 0.005 Hz sine wave is shown, neither displaying any significant signs of phase lag. Figure 17 shows the result for the 0.02 Hz sine wave, where a 0.3 s phase lag can be identified.

**Figure 18:**
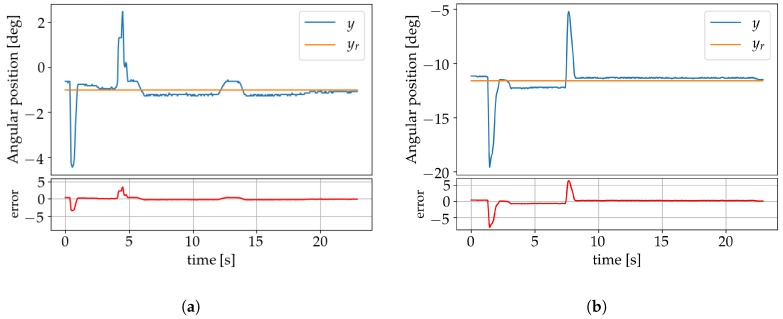
Plots showing the results when applying an extra: (**a**) 85% torque; (**b**) 143% torque.

The effect of disturbances on the system was investigated by a sudden application of additional torque on the arm that was removed once the system had stabilized. This was done first by applying an extra 86% of torque (Figure 18a), and later an extra 143% (Figure 18b). In both cases, the perturbation was compensated for by the controller without additional learning to adjust the weights.

## 5. Conclusions

A real-time, low computational cost, neural network direct control, with online learning, was developed for position control of shape memory alloy manipulators. Neural network weight coefficients were updated online by using the sensor position data while the controller was applied to the system, without previous training of the neural network weights nor the inclusion of a hysteresis model. Experimental evaluation was performed on a 1-DOF manipulator system actuated by a shape memory alloy wire and test results verify the effectiveness of the proposed control scheme to control the system angular position, compensating for the hysteretic behavior of the shape memory alloy actuator. Using a learning algorithm based on sending the actuator to distinct set-points within the possible range, a maximum static error of 1.28° was achieved when validating it against a staircase reference signal. This was later improved upon using a 0.01 Hz sine wave reference signal for learning, resulting in a maximum error of 0.83° for the same type of validation, or 0.49° for set-points above −12°. The effectiveness of the controller was also tested against a 0.04 Hz ramp and sine waves of frequencies 0.005-, 0.01- and 0.02 Hz without showing any severe signs of phase lag, with the worst case (0.02 Hz) presenting a lag of 0.3 s. The controller also showed its ability to compensate for load disturbances, when an additional torque of 143% was applied.

For future work, a detailed comparison with results for other existing techniques could be performed, including changing the control system and neural network architecture, as well as the utilized learning algorithm. In addition, a study of the effect of periodic disturbances on the system could be performed, as well as an investigation into if the system can be controlled without the use of sensors.

## Figures and Tables

**Table 1:** Flexinol wire characteristics.

Model No.	Diameter	Length	Operational Current	Transition Temperature	Pull Force	Resistance
STD-005-90	0.13 mm	305 mm	200 mA	90°	0.22 kg	0.75 Ω/cm

**Figure A1:**
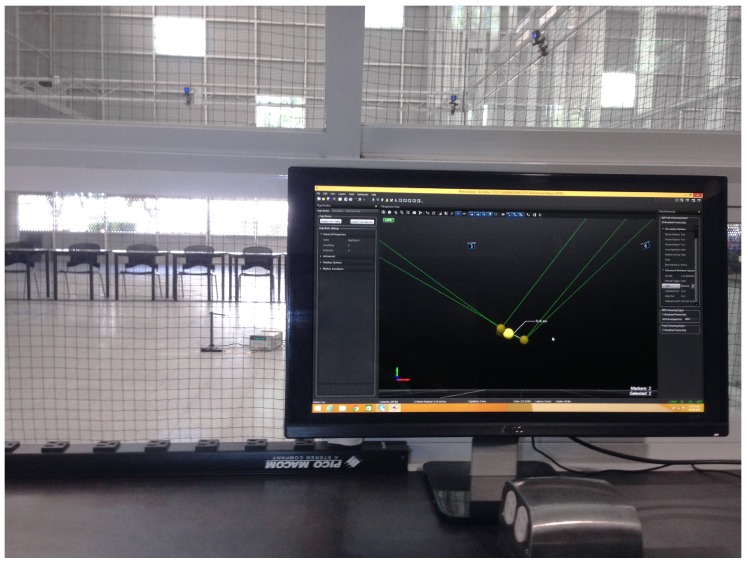
The setup of the motion capture system.

**Figure A2:**
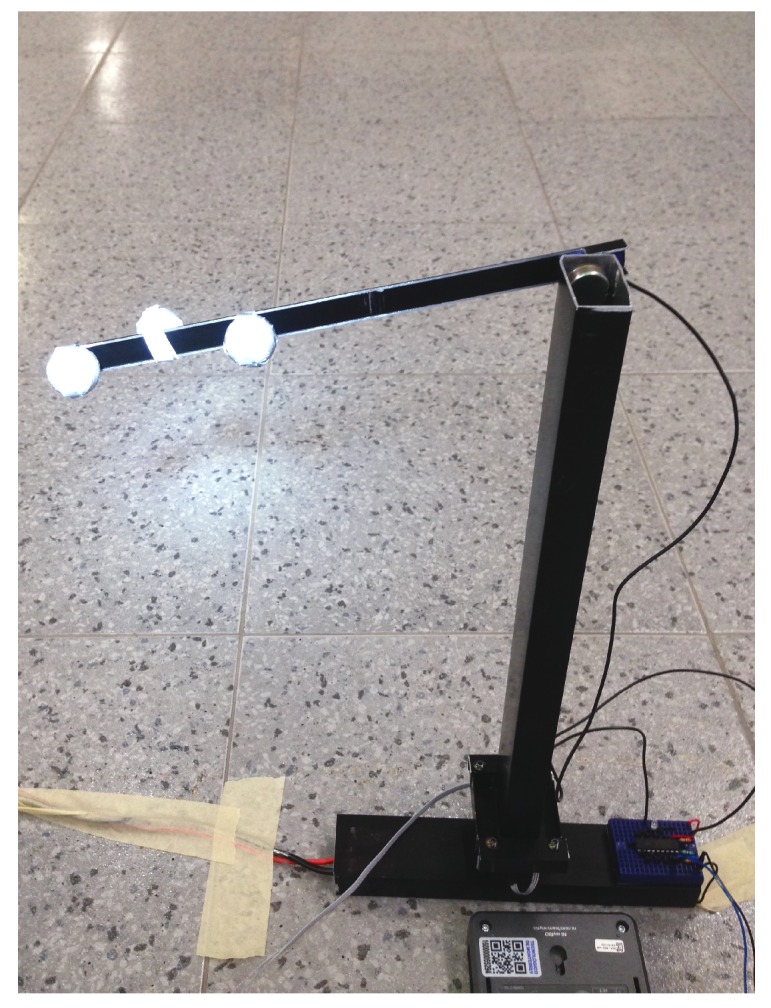
Setup of the reflective markers.

**Figure A3:**
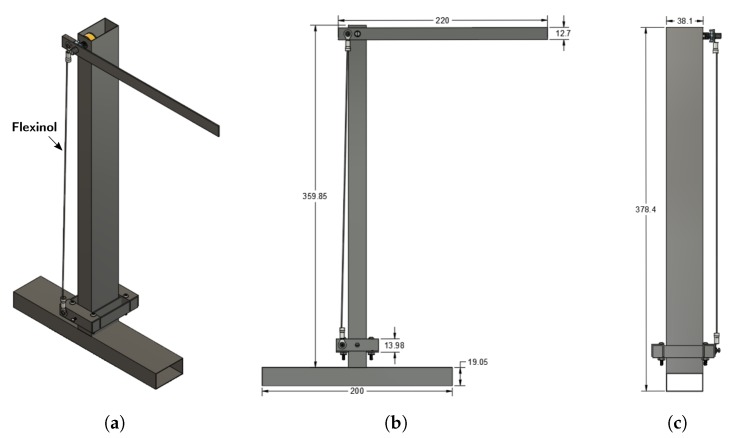
Schematic showing the dimensions in millimeters of the 1-DOF manipulator used in experiments: (**a**) Perspective view; (**b**) Front view; (**c**) Side view.

## Abbreviations

The following abbreviations are used in this manuscript:

(A)NN	(artificial) neural network
1-DOF	one degree of freedom
MEMS	micro-electromechanical systems
MLP	multilayer perceptron
PID	proportional-integral-derivative
PWM	pulse-width modulation
SMA	shape memory alloy

## Appendix A. Motion Capture System

The setup of the motion capture system is seen in Figure A1 and Figure A2 and resulted in the calibration equation

(A1) y=53x−128,

with a linear correlation coefficient R=0.9997, where *y* is the angular position and *x* the potentiometer voltage.

## Appendix C. Back-Propagation

**Table:** **Listing 1:** The back-propagation algorithm in pseudocode.

# SETUP define vector "inputs" = [x_1,...,x_n] define vector "correct_outputs" = [d_1,...,d_n] define boolean "DONE" = FALSE initialize float "eta" > 0 initialize float "tolerance" > 0 initialize random weights "w_ij" and "v_j" initialize float "t" > 0 # TRAINING while not DONE: | for x_k in inputs and d_k in correct_outputs: | | # Calculate hidden layer activation | | h_j = sigmoid( sum_over_i(x_i∗w_ji) ) | | | | # Calculate output layer activation | | u = sigmoid( sum_over_j(h_j∗v_j) ) | | | | # Let the system respond | | # before calculating error | | wait t seconds | | | | # Calculate error | | e_y_k = x_k-d_k | | | | # Update weights | | w_ji += eta∗e_y_k∗u∗(1-u)∗h_j | | v_j += eta∗e_y_k∗u∗(1-u)∗h_j∗(1-h_j)∗v_j∗x_i | end for | | if e_y_k < tolerance for all k: | DONE = TRUE end while
